# Supplementary material for: Understanding the Immunomodulatory Effects of Bovine Colostrum: Insights into IL-6/IL-10 Axis-Mediated Inflammatory Control
Source: Vet Sci. 2023 Aug 11;10(8):519. doi: 10.3390/vetsci10080519 (PMC10458264; doi:10.3390/vetsci10080519)
Supplement: Supplementary file 1 [file vetsci-10-00519-s001.zip › vetsci-2539353-supplementary.pdf]

Article

Understanding the Immunomodulatory Effects of Bovine Colostrum: Insights into IL-6/IL-10 Axis-Mediated Inflammatory Control

Ramunė Grigalevičiūtė [1,2,*], Paulius Matusevičius [2], Rita Plančiūnienė [3], Rolandas Stankevičius [2], Eivina Radzevičiūtė-Valčiukė [4], Austėja Balevičiūtė [5], Augustinas Želvys [4], Auksė Zinkevičienė [4], Vilma Zigmantaitė [1], Audrius Kučinskas [1] and Povilas Kavaliauskas [1,6,7,8,*]

1 Biological Research Center, Lithuanian University of Health Sciences, Tilzes Str. 18/7, LT-47181 Kaunas, Lithuania; vilma.zigmantaite@lsmuni.lt (V.Z.); audrius.kucinskas@lsmuni.lt (A.K.)
2 Department of Animal Nutrition, Lithuanian University of Health Sciences, Tilzes Str. 18, LT-47181 Kaunas, Lithuania; paulius.matusevicius@lsmu.lt (P.M.); rolandas.stankevicius@lsmu.lt (R.S.)
3 Institute of Microbiology and Virology, Lithuanian University of Health Sciences, Eiveniu Str. 4, LT-50161 Kaunas, Lithuania; rita.planciuniene@lsmu.lt
4 Centre for Innovative Medicine, Department of Immunology, Santariskiu Str. 5, LT-08410 Vilnius, Lithuania; eivina.radzeviciute@gmail.com (E.R.-V.); augustinas.zelvys@gmail.com (A.Z.); aukse.zinkeviciene@googlemail.com (A.Z.)
5 Institute of Environmental Medicine, Toxicology Unit, Karolinska Institutet, Stockholm, Solnavägen 1, 17177 Solna, Sweden; baleviciuteausteja@gmail.com
6 Joan and Sanford I. Weill Department of Medicine, Weill Cornell University, 1300 York Avenue, New York, NY 10065, USA
7 Department of Microbiology and Immunology, University of Maryland Baltimore School of Medicine, Baltimore, MD 21201, USA
8 Institute of Infectious Diseases and Pathogenic Microbiology, Birstono Str. 38A, LT-59116 Prienai, Lithuania
* Correspondence: ramune.grigaleviciute@lsmuni.lt (R.G.); pok4001@med.cornell.edu (P.K.)

Figure S1. The full length Western blot immage demonstrating human total NF-κB and beta-actin in THP-1 derived macrophages as described in materials and methods section of the manuscript.

Figure S2. The full length Western blot immage demonstrating human total NF-κB and beta-actin in Caco-2/THP-1 co-culture as described in materials and methods section of the manuscript.

Figure S3. The full length Western blot immage demonstrating human phosphor-NF-κB (Ser536) and beta-actin in THP-1 macrophages as described in materials and methods section of the manuscript.

Figure S4. The full length Western blot immage demonstrating human phosphor-NF-κB (Ser536) and beta-actin in Caco-2/THP-1 co-culture as described in materials and methods section of the manuscript.

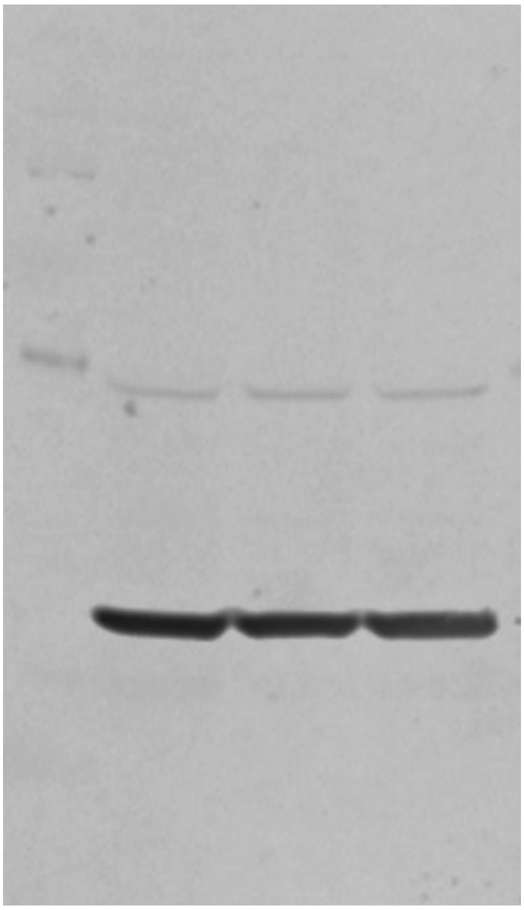

Figure S1. The full length Western blot immage demonstrating human total NF-κB and beta-actin in THP-1 derived macrophages as described in materials and methods section of the manuscript.

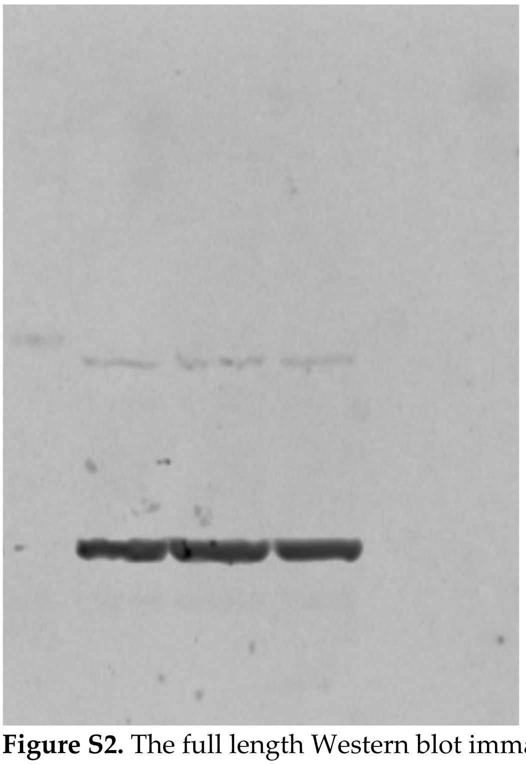

Figure S2. The full length Western blot immage demonstrating human total NF-κB and beta-actin in Caco-2/THP-1 co-culture as described in materials and methods section of the manuscript.

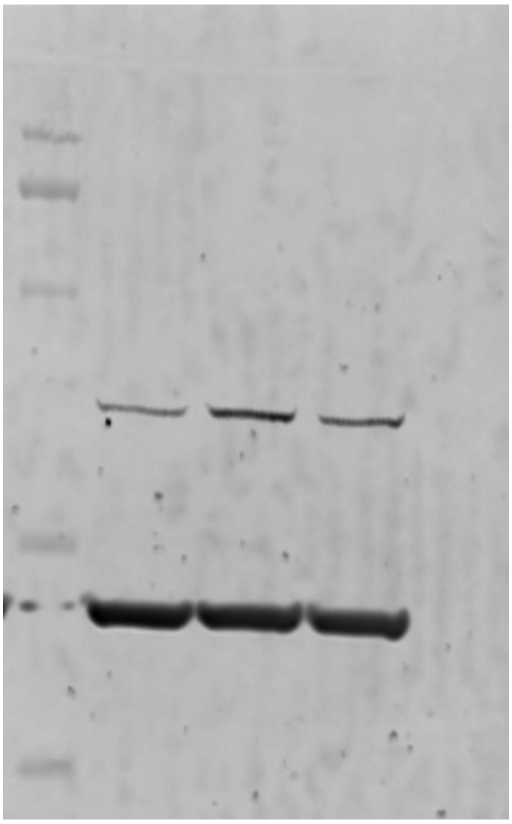

Figure S3. The full length Western blot immage demonstrating human phosphor-NF-κB (Ser536) and beta-actin in THP-1 macrophages as described in materials and methods section of the manuscript.

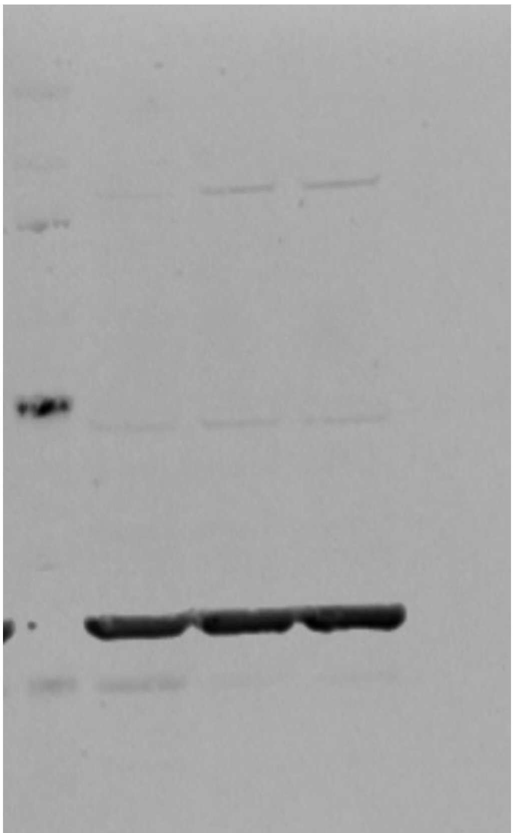

Figure S4. The full length Western blot immage demonstrating human phosphor-NF-κB (Ser536) and beta-actin in Caco-2/THP-1 co-culture as described in materials and methods section of the manuscript.
